# Evaluating Telehealth Adoption and Related Barriers Among Hospitals Located in Rural and Urban Areas

**DOI:** 10.1111/jrh.12534

**Published:** 2020-11-12

**Authors:** Jie Chen, Aitalohi Amaize, Deanna Barath

**Affiliations:** Department of Health Policy and Management, School of Public HealthUniversity of Maryland, College Park, Maryland

**Keywords:** health information exchange, health information technology, patient engagement, rural health, telehealth

## Abstract

**Purpose:**

To assess telehealth adoption among hospitals located in rural and urban areas, and identify barriers related to enhanced telehealth capabilities in the areas of patient engagement and health information exchange (HIE) capacity with external providers and community partners.

**Methods:**

We used the 2018 American Hospital Association (AHA) Annual Survey and IT Supplement Survey. We applied state fixed effects multivariate analyses and Oaxaca decomposition to estimate the variation of outcomes of interest by hospital geographies.

**Findings:**

Our research showed substantial differences in telehealth adoption among hospitals located in rural, micropolitan, and metropolitan areas, where adoption rates increase with urbanicity. Rural hospitals were least likely to have telehealth systems with patient engagement capabilities such as the ability to view their health information online and electronically transmit medical information to a third party. They were also the least likely to report that clinical information was available electronically from outside providers. Our model explained 65% of the rural/urban difference in telehealth adoption, 55% of the number of telehealth services adopted, and 43%-49% of the rural/urban difference in telehealth barriers.

**Conclusion:**

Findings demonstrated significant barriers to telehealth use among hospitals located in rural and urban areas. For rural hospitals, barriers include lack of HIE capacity among health care providers in the community, and lack of patient engagement capability.

According to the Office of Management and Budget, approximately 46 million people, or 15% of the population as of the 2010 Census, resided in rural areas, while over 72% of the US land area was designated as rural (nonmetropolitan).^1^ Compared to urban areas, rural areas have a higher percent of older adults,^2^ higher incidence of health disparities,^3,4^ lower patient volume in health care facilities, and substantially poorer health care infrastructure,^5,6^ including telehealth adoption and health information technology (HIT) system capabilities.^7,8^ Telehealth refers to a broad variety of technologies and tactics to deliver care, patient education, and support services virtually (see Appendix A).^9^ While telehealth can improve health care delivery, quality, and costs,^10,11^ the main barrier to telehealth adoption in rural hospitals is the cost of implementation,^12^ which could be exacerbated by the complicated and slow reimbursement for telehealth services.^13–15^ Other barriers include technologic concerns and a belief that patient needs can be met without telehealth.^12,13^

Telehealth services are enhanced by the capabilities of accompanying HIT systems, which can help coordinate care across the continuum, improve patient outcomes, and reduce health care costs.^10,11,16,17^ HIT systems refer to health information exchanges (HIE), electronic health records (EHR), and data collection, which can support care delivery by providing varying degrees of coordination and system integration.^10,18^ A priority of the Office of the National Coordinator for Health Information Technology is to leverage the capabilities of HIT systems to accelerate health care integration and invest in rural HIT infrastructure.^19^ Both horizontal multisector integration (across health care providers, home health agencies, nursing homes, long-term care facilities, community partners, public health systems, housing providers, transportation services, etc) and vertical-level integration (across programs, local networks and health systems, state health systems, and federal policies) are needed to improve treatment efficiency, population health, and health equity, particularly during the current ongoing pandemic.^11,17,20–25^ Enhancing HIE capabilities to include and engage more partners and improve data availability can facilitate care coordination through horizontal and vertical integration across multiple sectors.^11,16,26^

While the potential of utilizing telehealth to coordinate care is dependent upon the HIT infrastructure, patient engagement with the HIT system is also an important component. Overall, patient engagement is critical to achieve health care quality (ie, early diagnosis, continuity of care, treatment adherence) and reduce health disparities.^27,28^ Patient engagement with HIT systems, particularly EHRs and schedulers, promote self-management and health literacy.^8,29^ A recent study found that urban and rural providers used EHRs at similar rates, but patient engagement was lower among rural residents; authors of the study determined that this was due to variations in Internet access, access to a usual source of care, and whether there was provider encouragement to access records.^8^

The COVID-19 pandemic has advanced the way the health care industry utilizes telehealth.^30^ Evidence suggests telehealth is a useful tool to expand health care access by utilizing remote providers, streamlining treatment, and easing burnout among frontline health care providers.^26^ More importantly, the HIE functionalities of HIT systems are critical to the digitalization of health care, as it relates to interoperability, data management and integration, building predictive tools, surveillance systems, artificial intelligence, and postdischarge remote patient monitoring.^16,31–33^ Patient engagement with HIT systems is critical for scheduling appointments, ordering prescription refills, and accessing their health information if they need to see a new provider. Currently, rural areas are experiencing rapid rates of cases and deaths from COVID-19, which could be especially concerning given the potential to severely exacerbate pre-existing health care system-level challenges.^7,8,34^ Now is a critical time to understand barriers to implementing robust and responsive HIT systems.^29,35^ Such evidence is needed to inform an improved HIT infrastructure for rural populations.

Ultimately, telehealth can enhance care coordination by leveraging HIT capabilities to enable communication through EHRs and support patient-centered care. The objective of this study is to provide a baseline assessment of telehealth adoption among hospitals located in rural and urban areas and identify barriers to enhanced telehealth use in 2 areas: (1) patient engagement capabilities with HIT systems and (2) HIE capabilities with external providers and partners. We hypothesize that significant barriers exist, and modifiable factors can be identified to increase telehealth adoption and maximize the use of existing telehealth systems. We expect that results can be helpful to future evaluations of telehealth capacity-building efforts to improve the health of people residing in rural areas.

## Methods

### Data

This was a hospital-level analysis using the 2018 American Hospital Association (AHA) Annual Survey (n = 4,608), an annual census of US hospitals with about an 80% response rate that captures hospital structure, facilities, services, personnel, and spending.^9^ This study focused on general medical and surgical hospitals that responded to the telehealth section to measure telehealth adoption (n = 3,537). We further linked the AHA Annual Survey with data from the 2018 AHA Annual Information Technology (IT) Supplemental Survey to track in-depth measures of barriers to telehealth capabilities (n = 2,277). The AHA IT survey was administered from January 2019 to April 2019 and captures IT capabilities, interoperability, HIE barriers, and patient engagement with HIT systems.^36^

### Measures

#### Telehealth Adoption

Telehealth is defined by the AHA as a “broad variety of technologies and tactics to deliver virtual medical, public health, health education delivery and support services using telecommunications technologies. Telehealth is used more commonly as it describes the wide range of diagnosis and management, education, and other related fields of health care. This includes, but is not limited to: dentistry, counseling, physical and occupational therapy, home health, chronic disease monitoring and management, disaster management and consumer and professional education.”^9^ Telehealth adoption was first assessed across hospitals. The following hospital-level telehealth measures were reported in the Facilities and Services section of the AHA Annual Survey: consultation and office visits; eICU; stroke care; psychiatric and addiction treatment; remote patient monitoring: postdischarge, ongoing chronic care management, and other remote patient monitoring; and other telehealth services.^9^ We created telehealth adoption indices to track any use of telehealth services and number of services adopted, if any (range from 1 to 8). Different measures were tested in the sensitivity analysis.

#### Patient Engagement and Health Information Exchange Capabilities—Barriers to Enhanced Telehealth Use From the AHA IT Supplement Survey

First, we examined the patient engagement capabilities with hospital HIT systems. Hospitals reported whether patients treated in the hospital were able to: (1) view their health/medical information online, (2) download information from their medical record, (3) import their medical records from other organizations into their portal, (4) electronically transmit (send) medical information to a third party, (5) request an amendment to change/update their medical record, (6) request refills for prescriptions online, (7) schedule appointments online, (8) pay bills online, (9) submit patient-generated data, (10) communicate via secure messaging with providers, (11) designate proxy access, (12) view clinical notes online, and (13) access medical information using applications configured to meet the application programming interfaces (API) specifications in the EHR. We created an index which equaled the summation of these 13 indicators.

Next, we examined HIE capabilities with external providers and partners as it requires integrated and compatible HIT systems across users (partners, hospitals, and outside providers).^16,19,29^ Hence, we assessed 3 measures related to HIE capabilities: (1) providers able to query electronically for patient health information from sources outside ( = 1 [yes] or = 0 [no]); (2) clinical information available electronically from outside providers ( = 1 [yes] or = 0 [no]); and (3) frequency of use of electronic patient health information from outside providers ( = 1 [often/sometimes] or = 0 [rarely/never]).

#### Rural, Micro, and Urban Hospitals

Hospital geographic categories are defined by the Core-Based Statistical Areas (CBSAs) used by the US Office of Management and Budget and obtained from the AHA Annual Survey.^1^ The 3 categories provided are metropolitan (metro), micropolitan (micro), and rural. A metro CBSA has at least 1 core urbanized area of 50,000 or more people that is adjacent to a territory with a high degree of social and economic integration with the core, measured by commuting ties. A micro CBSA is made up of urban clusters with at least 10,000 people but less than 50,000 people. Lastly, rural refers to areas outside of the defined urban (metro and micro) CBSAs.

#### Other Covariates

Covariates include various hospital-level characteristics. Hospital-level characteristics capture organizational factors such as: control type (not-for-profit, for-profit, or government), bed size (small if <50 beds, medium if 50-200 beds, large if ≥200 beds), and safety-net status (defined = 1 if Medicaid claims > mean of the state +1 standard deviation; 0 otherwise^37^).

### Study Design

This study design followed the multilevel approach used in the study by Chen and associates from 2018 which examined variations in hospital adoption of care coordination services.^38^ We first summarized hospital-reported telehealth adoption and related barriers across hospitals in rural, micro, and metro geographies and used a *t*-test to compare differences in rural and micro areas to metro areas. We then used univariate analysis to estimate the variation in outcomes of interest by hospitals’ metro, micro, or rural status (Model 1). Outcomes were telehealth adoption, and barriers and challenges to telehealth capabilities. The estimation model was expanded to include hospital level (Model 2) associated with telehealth capabilities. Different estimation strategies were applied for different model specifications. Multivariate logistic regressions were applied to examine geographic differences in (1) any telehealth adoption, (2) providers’ ability to query electronically for patient health information from sources outside, (3) availability of clinical information electronically from outside providers, and (4) whether hospitals often/sometimes use electronic patient health information from outside providers. Ordinary least squares regressions were applied to examine geographic differences in (1) number of telehealth services adopted, and (2) index score of patient engagement capabilities.

We applied state fixed effects estimation in all the analyses and reported marginal effects for all the covariates. Finally, we implemented the Oaxaca decomposition to examine whether variations in hospital characteristics can explain hospital rural and urban disparities in the adoption of telehealth and related barriers.^39^

We tested different model specifications and applied different estimation strategies as sensitivity analyses. For the telehealth adoption measures, we derived 2 broad service types divided into further categories: (1) any use of telehealth services (consultation and office visits, eICU, stroke care, psychiatric and addiction treatment, other telehealth) and the number of telehealth services adopted, if any (1-5);^40^ and (2) any use of telehealth remote patient monitoring services (postdischarge, ongoing chronic care management, and other remote patient monitoring) and the number of remote patient monitoring services adopted, if any (1-3). We also applied factor analysis, and results (specifying eigenvalue at 1) showed 2 factors; one was associated with telehealth remote patient monitoring, and the other was associated with telehealth services. Results were consistent with the overall combined telehealth adoption indicator and are available upon request. Finally, we also tested different sets of county-level characteristics (eg, demographic composition, poverty level, home health agencies, community mental health centers, and FQHCs, using the 2018 Area Health Resource File^41^), yielding similar results. All analyses were performed using STATA 16 MP 4 (StataCorp LLC, College Station, TX).

## Results

The top panel of Table 1 presents hospital-level telehealth adoption by rural, micro, and metro area geographies. Compared to hospitals located in metro areas, hospitals in rural areas were significantly less likely to adopt any of the 8 telehealth services (0.54 vs 0.75, *P* < .001) and adopted fewer telehealth services, if any (1.98 vs 2.72, *P* < 0.001). All telehealth services were adopted significantly less in rural areas compared to metro areas. Particularly, metro area hospitals were more than twice as likely as rural area hospitals to adopt telehealth eICU, stroke care, psychiatric and addiction treatment, and other remote patient monitoring unrelated to ongoing chronic care management. They were also more than 3 times as likely to adopt telehealth remote patient monitoring postdischarge. Similar trends were observed among hospitals located in micro area hospitals compared to metro area hospitals. Compared to metro area hospitals, hospitals located in micro areas were significantly less likely to adopt any telehealth services and adopted fewer services, if any. The rates of adoption of each individual telehealth service for micro area hospitals were between the adoption rates of rural area and metro area hospitals.

The bottom panel of Table 1 presents the barriers of enhanced telehealth use in patient engagement capabilities and HIE capabilities. Metro area hospitals had telehealth systems with the highest patient engagement capabilities, followed by micro area and rural area hospitals. In other words, patients served in metro area hospitals were more likely to have access to telehealth systems that enabled more patient engagement activities, including the ability to view their health information online, electronically transmit medical information to a third party, and more. Among specific patient engagement measures, results showed that compared to hospitals located in metro areas, hospitals located in rural areas were more likely to report their patients’ inability or difficulty to view their medical record online, electronically transmit medical information to a third party, request an amendment to change/update their medical record, request refills for prescriptions online, schedule appointments online, pay bills online, submit patient-generated data, and communicate via secure messaging with providers.

Similar differences in HIE capacity were found. Hospitals located in metro areas were more likely to report that clinical information was available electronically from outside providers, followed by hospitals located in micro areas and rural areas (0.67 vs 0.50, *P* < .001; 0.67 vs 0.46, *P* < .001). Similarly, the measures of providers’ ability to query electronically for patient health information from an outside source, and frequency of use of electronic patient health information from outside providers were significantly higher in hospitals located in metro areas, followed by hospitals located in micro areas. These scores were lowest among rural area hospitals. Consistent with the literature, compared to rural area hospitals, hospitals located in urban areas had significantly larger bed size and were more likely to be a for-profit hospital (results not shown).

Table 2 presents univariate regression results (Model 1), and results after controlling for hospital characteristics (Model 2). We applied the state fixed effects estimation to all the regressions to account for state-level variation in policies, among others. Model 1 demonstrated significant urban and rural disparities in telehealth adoption and reported barriers. The likelihood of adopting any telehealth, and the number of telehealth services adopted were significantly lower in rural area and micro area hospitals, compared to metro area hospitals. Measures of telehealth patient engagement capabilities and capabilities for patient HIE with outside providers were significantly lower in rural area hospitals and micro area hospitals compared to metro area hospitals.

After controlling for hospital characteristics (Model 2), results showed that rural area hospitals were still significantly less likely to adopt telehealth services overall, and that they adopted fewer telehealth services compared to metro area hospitals; such differences were no longer significant among micro area hospitals. Compared to metro area hospitals, rural hospitals were 6% less likely to adopt any telehealth services (95% CI: −0.10 to −0.02), and they faced significantly substantial telehealth barriers related to patient engagement capabilities and HIE capabilities; these measures were also significantly lower in micro area hospitals except the patient engagement capabilities score.

Based on the state fixed effects estimation of Model 2, we applied the decomposition approach to predict the likelihood of telehealth adoption, the number of adopted telehealth services, and reported telehealth barriers between hospitals located in rural and metropolitan or urban areas (Table 3). Results demonstrated significantly lower levels of telehealth adoption and poorer telehealth capabilities in rural area hospitals overall, compared to urban area hospitals. Our model explained 65% of the rural/urban difference in any telehealth adoption, 55% of the number of telehealth services adopted, and 43%-49% of the rural/urban difference in telehealth patient engagement and HIE capabilities. Small bed sizes of rural hospitals were one of the major factors that predicted the urban and rural differences in telehealth adoption as well as other measures.

## Discussion

Our study explored the differences in hospital telehealth adoption in rural and urban areas. Findings support current research demonstrating substantial differences in telehealth adoption across hospitals located in rural, micro, and metro areas.^7,12^ Our contribution to this literature is the adoption of specific telehealth services across geographies. Results also suggested that hospitals located in rural areas were less likely to have telehealth systems capable of facilitating robust patient engagement compared to metro and micro areas. The capabilities around scheduling appointments online, requesting prescription refills, submitting patient generated data, viewing clinical notes, and accessing medical information using online applications varied across geographies, with the ability to import medical records utilized the least across geographies. We speculate that during the COVID-19 pandemic and in its aftermath, robust, user-friendly telehealth systems in rural areas can facilitate patient engagement, especially for people with complex health needs, though training would be needed for both patients and health care providers to encourage frequent use of HIT systems.^42^

Our study also explored barriers to enhance telehealth use in HIE capabilities with external providers and partners. The results of our study showed substantial variation in HIE capabilities across geographies: rural hospitals were more likely to report lacking capabilities to query patient health information from outside sources and routine electronic access to patient clinical information from outside sources, and therefore utilized electronic patient health information from outside provides less often, if at all, compared to metro area hospitals. Micro hospitals had the same pattern with results greater than rural hospitals. HIE requires an interoperable infrastructure to enable communication and information-sharing between health care providers across multiple settings, and among community partners from health or social services sectors.^16,19,29,43^ Such systems can promote more efficient data exchange, tracking, and integration. Our results support the urgent need to build and strengthen existing integrated data systems in rural areas.^44^

Finally, study results showed that substantial differences in telehealth use between rural and urban hospitals could be explained by our model specifications. Hospital bed size and control type could explain a substantial part of urban and rural differences, consistent with the literature on health care disparities in urban and rural hospitals.^7,8^ We also note that there was still a substantial portion of geographic differences in telehealth adoption and related barriers that was not explained by hospital- and state-level characteristics.

Unobserved factors not captured in the model could relate to the real demand for health care and telehealth care. It is likely that compared to urban areas, there was lower overall demand in rural areas due to lower population density. However, research has shown that, on average, rural residents have poorer health status relative to urban residents.^45,46^ This indicates that there is room for improvement in leveraging telehealth to meet the demand for better health among rural residents.^47^ Demand can be generated by improving knowledge about the potential for telehealth to improve care access, by increasing familiarity with telehealth functionality, and by training and educating rural residents and health care providers on telehealth use.^10^

Unobserved factors could also relate to hospital and hospital system characteristics that we were not able to measure in this analysis. In addition, unobserved factors could relate to measures of the quality of hospital telehealth systems, which were not available. More detailed measures are needed at the HIT infrastructure level, such as the comprehensiveness of data, timeliness of data transfer/exchange, data integration across multiple health and social sectors, privacy and security, reporting burden, and ease of use.^43^

This study has several limitations. First, our data were subject to measurement error and recall bias. Hospitals that responded to the AHA IT survey were more resourceful and well equipped. Even though our study still observed a significant difference in the adoption of telehealth and barriers to enhanced capabilities of HIT systems across geographies, we would imagine rural and urban differences would be more pronounced than we observed from the data. The AHA Annual Survey itself is representative and its response rate reached 80% and higher. However, the AHA IT supplementary survey may or may not reflect the nationally representative statistics. Second, the goal of the study was to provide a baseline assessment of telehealth use among hospitals in various rural and urban areas. Future studies can further explore rural-urban variations through specific characteristics, such as hospital organizational, service area, and market density measures. Such measures can help capture the unobserved factors that we were not able to measure in this study. Third, this was a hospital-level analysis. Future analysis can be applied to hospitals that provide different services, such as psychiatric services, long-term care services, etc. Future studies may also include patient-level information on health needs and social determinants of health. The combined analysis may further suggest a need for more patient-centric telehealth systems designed for people with different health needs and social determinants of health. Additionally, HIE capabilities did not account for hospitals acting as hubs or spokes for the exchange network.^48^

We sought to provide a baseline assessment of telehealth use among hospitals located in rural, micro, and metro areas. The next step is to assess specific telehealth services and estimate their impact on specific populations in need. For example, estimating the impact of tele-behavioral health services, such as the use of telehealth psychiatric and addiction treatment, on mental health screening and treatment, as rural residents face substantial barriers in accessing mental health care. Future studies should also examine the mechanisms by which telehealth can improve multisector care coordination, health care quality, and health outcomes among residents of rural areas. Such evidence will be helpful to inform policy interventions that incentivize, encourage, and expand the use of telehealth among health care providers and patients.

## Policy Implications

Results of this study demonstrated disparities in telehealth adoption and capacity among hospitals located in rural areas, compared to those located in urban areas. Barriers identified can provide evidence on how to strengthen telehealth systems in rural areas in the future. The COVID-19 pandemic is having a major impact and will change the landscape of telehealth use in the long run.^30^ As more investments are directed toward developing and strengthening the telehealth capacity in rural areas (including access to broadband networks), our analysis aims to provide important baseline evidence on current hospital telehealth use and barriers.^49^

It will be more important than ever to understand the concepts of population health in the post COVID-19 era. Effective and efficient prevention and treatment requires integration across systems. Data communication among diverse stakeholders thus becomes extremely critical to promoting care coordination. Ongoing policy initiatives such as the Accountable Care Organization (ACO) alternative payment model and the ACO Investment Model (AIM) are designed to promote care coordination through financial incentives.^15,50–56^ During emergency crisis situations, such initiatives should be adjusted to reimburse for quality care. By encouraging the use of telehealth, payment models can be designed in such a way as not to add undue financial burden on health care providers.^57^

The COVID-19 crisis has underscored the critical role of local health departments in advancing population health.^22^ Future research should evaluate how the public health system can lead or facilitate care coordination efforts across multiple sectors to advance health for rural residents, and whether telehealth systems can play a major role by enabling data communication, exchange, and sharing across public health systems.^18,58^ Meanwhile, payment design policies and financing systems should be evaluated to determine their effectiveness in promoting integrated and interoperable data systems.^57^ It will also be important to research the cost-effectiveness of public health-driven telehealth capacity-building efforts.

## Conclusion

This study demonstrated significant disparities in telehealth use and related barriers among hospitals located in rural and urban areas. Results also noted that barriers to widespread telehealth use in rural hospitals included lack of HIE capacity among external health care providers in the community and lack of telehealth capacity to facilitate patient engagement. Results provide baseline data upon which to examine the importance of promoting data communication, exchange, and sharing among multiple sectors, and to advocate for policy design to encourage telehealth capacity building to improve rural health.

## Figures and Tables

**Table 1:** Telehealth Adoption and Related Barriers Reported by Hospitals Located in Rural, Micro, and Metro Areas

	Metropolitan Areas			Rural Areas				Micropolitan Areas			
			
	n	Mean	SD	n	Mean	SD	*P* value	n	Mean	SD	*P* value
**Adoption of any telehealth (Yes/No)**	2,156	0.75	0.43	781	0.54	0.50	.00	600	0.64	0.48	.00
**Number of telehealth services adopted if any (an index from 1-8; the summation of the following 8 individual dichotomous (Yes/No) measures)**	1,617	2.72	1.83	420	1.98	1.19	.00	383	2.27	1.43	.00
Telehealth consultation and office visits	2,156	0.33	0.47	781	0.30	0.46	.04	600	0.32	0.46	.40
Telehealth eICU	2,156	0.48	0.50	781	0.13	0.34	.00	600	0.24	0.43	.00
Telehealth stroke care	2,156	0.43	0.49	781	0.21	0.40	.00	600	0.33	0.47	.00
Telehealth psychiatric and addiction treatment	2,156	0.20	0.40	781	0.10	0.30	.00	600	0.16	0.36	.02
Telehealth remote patient monitoring: postdischarge	2,156	0.14	0.35	781	0.03	0.18	.00	600	0.08	0.28	.00
Telehealth remote patient monitoring: ongoing chronic care management	2,156	0.18	0.38	781	0.12	0.33	.00	600	0.13	0.34	.02
Telehealth other remote patient: monitoring	2,156	0.13	0.34	781	0.05	0.21	.00	600	0.05	0.22	.00
Other telehealth	2,156	0.15	0.36	781	0.13	0.33	.02	600	0.15	0.36	.06
**Patient engagement capabilities (an index from 1-13; the summation of the following 13 individual dichotomous (Yes/No) measures)**	1,517	9.72	2.94	400	8.42	3.27	.00	360	9.25	2.99	.01
View their health/medical information online	1,788	0.98	0.13	573	0.96	0.20	.00	479	0.98	0.13	.94
Download information from their medical record	1,763	0.94	0.24	557	0.90	0.30	.00	467	0.94	0.23	.75
Import their medical records from other organizations into your portal	1,729	0.40	0.49	518	0.38	0.48	.39	435	0.40	0.49	.78
Electronically transmit (send) medical information to a third party	1,722	0.80	0.40	527	0.66	0.47	.00	441	0.78	0.42	.22
Request an amendment to change/update their medical record	1,723	0.78	0.42	534	0.64	0.48	.00	453	0.75	0.43	.20
Request refills for prescriptions online	1,770	0.65	0.48	552	0.55	0.50	.00	464	0.63	0.48	.48
Schedule appointments online	1,783	0.71	0.45	558	0.45	0.50	.00	471	0.59	0.49	.00
Pay bills online	1,775	0.90	0.30	560	0.78	0.42	.00	474	0.89	0.31	.71
Submit patient-generated data	1,730	0.62	0.49	525	0.44	0.50	.00	448	0.49	0.50	.00
Secure messaging with providers	1,772	0.80	0.40	558	0.73	0.44	.00	473	0.75	0.43	.04
Proxy access	1,754	0.92	0.27	544	0.83	0.38	.00	465	0.90	0.30	.21
View clinical notes online	1,725	0.60	0.49	517	0.56	0.50	.11	449	0.59	0.49	.52
Access medical information using applications configured to meet the API specifications in your EHR	1,722	0.53	0.50	500	0.50	0.50	.31	442	0.52	0.50	.85
**Health information exchange capabilities (an index from 1-3; the summation of the following 3 individual dichotomous measures)**											
Providers able to query electronically for patient health info from outside sources (Yes/No)	1,676	0.80	0.40	512	0.56	0.50	.00	433	0.69	0.46	.00
Clinical information available electronically from outside providers (Yes/No)	1,726	0.67	0.47	524	0.46	0.50	.00	440	0.57	0.50	.00
Use of electronic patient health information from outside providers (Yes = Often/Sometimes, No = Rarely/Never)	1,695	0.73	0.44	502	0.58	0.49	.00	441	0.61	0.49	.00

Source: The 2018 AHA annual survey, 2018 AHA IT Supplement Survey.

Note: Hospital geographies are defined by core-based statistical areas, where rural is neither metro nor micro. Metropolitan was the reference group.

**Table 2:** Urban and Rural Differences in Adoption of Telehealth and Barriers of Information Exchange

	Any Telehealth^a^			# Telehealth Services if Any^b^			Patient Engagement Capabilities^b^			HIE Capability for Electronic Query for Patient Info From Outside Sources^a^			HIE Capability for Electronic Clinical Info Available From Outside Providers^a^			Frequency of Use of Electronic Patient Info From Outside Providers^a^		
						
MODEL 1	ME	95% CI		ME	95% CI		ME	95% CI		ME	95% CI		ME	95% CI		ME	95% CI	
Hospital Geography																		
Metro		Reference			Reference			Reference			Reference			Reference			Reference	
Rural	−0.19	−0.23	−0.16	−0.74	−0.94	−0.55	−1.53	−1.89	−1.18	−0.23	−0.27	−0.19	−0.22	−0.27	−0.17	−0.17	−0.22	−0.12
Micro	−0.11	−0.15	−0.07	−0.39	−0.58	−0.20	−0.71	−1.06	−0.36	−0.12	−0.16	−0.07	−0.11	−0.16	−0.06	−0.13	−0.18	−0.08
State fixed effect		Controlled			Controlled			Controlled			Controlled			Controlled			Controlled	
MODEL 2: Model 1 + hospital characteristics																		
Hospital Geography																		
Metro		Reference			Reference			Reference			Reference			Reference			Reference	
Rural	−0.06	−0.10	−0.02	−0.31	−0.54	−0.09	−0.75	−1.16	−0.35	−0.11	−0.16	−0.06	−0.12	−0.18	−0.07	−0.08	−0.13	−0.02
Micro	−0.03	−0.07	0.01	−0.06	−0.26	0.13	−0.27	−0.62	0.09	−0.05	−0.10	−0.01	−0.06	−0.11	−0.01	−0.08	−0.13	−0.03
Hospital Control																		
Not for profit		Reference			Reference			Reference			Reference			Reference			Reference	
For profit	−0.15	−0.20	−0.11	−0.64	−0.87	−0.40	−1.61	−2.01	−1.21	−0.10	−0.15	−0.04	−0.01	−0.07	0.05	−0.04	−0.10	0.01
Government	−0.08	−0.12	−0.04	0.29	0.11	0.47	−1.68	−2.00	−1.36	−0.18	−0.21	−0.14	−0.20	−0.25	−0.16	−0.18	−0.23	−0.14
Hospital bed size																		
Small		Reference			Reference			Reference			Reference			Reference			Reference	
Medium	0.10	0.07	0.14	0.18	−0.01	0.37	0.17	−0.16	0.50	0.07	0.03	0.11	0.01	−0.04	0.06	0.00	−0.05	0.04
Large	0.22	0.17	0.27	0.63	0.41	0.86	0.61	0.20	1.01	0.12	0.06	0.17	0.03	−0.02	0.09	0.05	−0.01	0.10
Safety-net status	0.11	0.05	0.18	0.60	0.39	0.80	0.73	0.34	1.11	0.09	0.02	0.15	0.12	0.06	0.18	0.10	0.04	0.16
State fixed effect		Controlled			Controlled			Controlled			Controlled			Controlled			Controlled	

a Ordinary least squares were applied.

b Results of logistic regressions.

Note: Model 1 was the univariate analysis; Model 2 = Model 1+ hospital characteristics. ME: marginal effects. 95% CI: 95% confidence interval. Hospital Control Types: Government category (including nonfederal: state, county, city, city-county, hospital district or authority; federal: department of defense, public health services, Veterans Affairs, Department of Justice, and other federal); nongovernment, not-for-profit category (church operated, other not-for-profit); for-profit category (individual-owned by individual, partnership, or corporation). Hospital bed size (small if <50 beds, medium if 50-200 beds, large if ≥200 beds). Safety-net status (defined = 1 if Medicaid claims > mean of the state+1 standard deviation; 0 otherwise).

**Table 3:** Oaxaca Decomposition to Explain Urban-Rural Difference in Hospital Telehealth Adoption and Related Barriers


	Any Telehealth				# Telehealth Services if Any				Patient Engagement Capabilities			
			
Differential	Coef	95% CI		%	Coef	95% CI		%	Coef	95% CI		%

Rural adoption	0.54	0.50	0.57		1.98	1.87	2.10		8.42	8.10	8.74	
Urban adoption	0.75	0.73	0.77		2.72	2.64	2.81		9.72	9.57	9.87	
Difference	−0.21	−0.25	−0.17		−0.74	−0.89	−0.60		−1.30	−1.66	−0.95	
Total explained rural vs urban difference	−0.14	−0.18	−0.10	**64.87%**	−0.41	−0.57	−0.24	**54.69%**	−0.57	−0.90	−0.23	**43.36%**
Individual factors	Coef	95% CI		%	Coef	95% CI		%	Coef	95% CI		%

For-profit hospital	0.02	0.01	0.02	−12.90	0.04	0.02	0.06	−9.29	0.18	0.12	0.24	−31.79
Government owned hospitals	−0.03	−0.04	−0.02	21.96	0.08	0.03	0.14	−20.67	−0.43	−0.56	−0.30	75.54
Bed size medium	−0.02	−0.02	−0.01	12.02	−0.03	−0.05	0.00	6.79	−0.03	−0.08	0.02	5.15
Bed size large	−0.11	−0.14	−0.08	78.81	−0.36	−0.48	−0.23	87.99	−0.36	−0.61	−0.11	63.67
Safety-net	−0.01	−0.02	0.00	9.13	−0.15	−0.21	−0.08	35.96	−0.16	−0.24	−0.08	28.39
State	0.01			−9.01	0.00			−0.79	0.23			−40.98
	Capability for electronic query for patient info from outside				Electronic clinical info available from outside				Frequency of use of electronic patient info from outside			
			
Differential	Coef	95% CI		%	Coef	95% CI		%	Coef	95% CI		%

Rural adoption	0.56	0.52	0.60		0.46	0.42	0.50		0.58	0.54	0.62	
Urban adoption	0.80	0.78	0.82		0.67	0.65	0.69		0.73	0.71	0.75	
Difference	−0.24	−0.29	−0.19		−0.21	−0.26	−0.16		−0.15	−0.20	−0.11	
Total explained rural vs urban difference	−0.12	−0.16	−0.07	**48.97%**	−0.10	−0.14	−0.05	**44.91%**	−0.07	−0.12	−0.02	**46.10%**
Individual factors	Coef	95% CI		%	Coef	95% CI		%	Coef	95% CI		%

For-profit hospital	0.01	0.00	0.02	−8.37	0.00	−0.01	0.01	−1.02	0.01	0.00	0.01	−7.10
Government owned hospitals	−0.06	−0.07	−0.04	49.09	−0.06	−0.08	−0.04	64.57	−0.05	−0.07	−0.03	70.38
Bed size medium	−0.01	−0.02	0.00	9.75	0.00	−0.01	0.00	3.15	0.00	−0.01	0.00	4.38
Bed size large	−0.08	−0.11	−0.04	64.74	−0.03	−0.07	0.00	31.60	−0.04	−0.08	−0.01	58.12
Safety-net	−0.01	−0.02	0.00	9.87	−0.02	−0.03	−0.01	21.16	−0.02	−0.03	0.00	22.86
State	0.03			−25.08	0.02			−19.46	0.03			−48.65


Note: Decomposes the telehealth disparities between rural and urban (metro & micro) hospitals. Main factors that contributed to urban and rural disparities were hospital-level characteristics.

## Appendix A: Definitions of Telehealth Measures

**Telehealth**. A broad variety of technologies and tactics to deliver virtual medical, public health, health education delivery and support services using telecommunications technologies. Telehealth is used more commonly as it describes the wide range of diagnosis and management, education, and other related fields of health care. This includes, but is not limited to: dentistry, counseling, physical and occupational therapy, home health, chronic disease monitoring and management, disaster management, and consumer and professional education.

- Consultation and office visits
- **elCU**. An electronic intensive care unit (eICU), also referred to as a tele-ICU, is a form of telemedicine that uses state of the art technology to provide an additional layer of critical care service. The goal of an eICU is to optimize clinical experience and facilitate 24-hour a day care by ICU caregivers.
- **Stroke care**. Stroke telemedicine is a consultative modality that facilitates the care of patients with acute stroke by specialists at stroke centers.
- **Psychiatric and addiction treatment**. Telepsychiatry can involve a range of services including psychiatric evaluations, therapy, patient education, and medication management.
- **Remote patient monitoring**. The use of digital technologies to collect medical and other forms of health data from individuals in one location and electronically transmit the information securely to health care providers in a different location for assessment and recommendation. Can be used for:
  - Postdischarge
  - Ongoing chronic care management
  - Other remote patient monitoring
